# A Scoping Review of Intimate Partner Violence Screening Programs for Health Care Professionals

**DOI:** 10.1371/journal.pone.0168502

**Published:** 2016-12-15

**Authors:** Sheila Sprague, Gerard P. Slobogean, Hayley Spurr, Paula McKay, Taryn Scott, Erika Arseneau, Muzammil Memon, Mohit Bhandari, Aparna Swaminathan

**Affiliations:** 1 Division of Orthopaedic Surgery, Department of Surgery, McMaster University, Hamilton, Ontario, Canada; 2 Department of Clinical Epidemiology and Biostatistics, McMaster University, Hamilton, Ontario, Canada; 3 Department of Orthopaedics, University of Maryland School of Medicine, Baltimore, Maryland, United States of America; 4 Graduate Entry Medicine, Royal College of Surgeons in Ireland, Dublin, Ireland; 5 Department of Family Medicine, McMaster University, Hamilton, Ontario, Canada; University of South Australia, AUSTRALIA

## Abstract

**Introduction:**

Between 38 and 59 percent of women presenting to health care professionals have experienced intimate partner violence. Consequently, multiple intimate partner violence identification or screening programs within health care settings have been developed; however, substantial variations in program content and interpretation of program effectiveness has resulted in conflicting practice guidelines. The purpose of our scoping review is to broadly identify and synthesize the available literature evaluating intimate partner violence identification programs within health care settings to identify key areas for potential evidence-based recommendations and to focus research priorities in the field.

**Materials and Methods:**

We conducted a search of MEDLINE, Embase, Cumulative Index of Nursing and Allied Health Literature, Cochrane Database of Systematic Reviews, Cochrane Central Register of Controlled Trials, and psycINFO. We used broad eligibility criteria to identify studies that evaluated intimate partner violence identification programs in health care settings. We completed all screening and data extraction independently and in duplicate. We used descriptive statistics to summarize all data.

**Results:**

We identified 59 eligible studies evaluating intimate partner violence identification programs within health care settings. The most commonly reported outcome themes were IPV disclosure (69%, n = 35), number of patients screened (39%, n = 20), HCP opinions towards screening (37%, n = 19), and patient opinions towards screening (29%, n = 15). The majority of studies (36 studies (70.6%)) reported positive program evaluation results.

**Discussion:**

The majority of studies reported positive program evaluation results. This may suggest that many different intimate partner violence identification programs are beneficial for identifying victims of abuse, however, it remains unknown as to whether identification programs prevent future episodes of abuse. Additionally, the substantial heterogeneity of the intervention characteristics, study methodology, and outcome measures assessed limits the ability to make clear recommendations as to the optimal method(s) of screening.

## Introduction

Intimate partner violence (IPV), also known as domestic violence, is a violation of human rights that disproportionately affects women. Between 38 and 59 percent of women presenting to health care professionals (HCPs) have experienced IPV [1], and it is estimated that on a global scale, one out of every three women experience IPV at some point in their lives [2]. IPV can escalate to intimate partner homicide, as evidenced by the fact that as many as 38 percent of all murders of women are perpetrated by intimate partners [3]. While women experiencing IPV visit their HCPs more frequently than women not experiencing IPV [4,5], many women are hesitant to disclose IPV, particularly when they are not specifically asked [6].

Taken together, these findings provide a strong rationale for optimizing health care settings to identify victims of IPV and initiate IPV services. Consequently, multiple programs within health care settings have been developed to identify women experiencing IPV; however, substantial variation in program content and effectiveness has created challenges in drawing conclusions [7–9]. Additionally, controversy exists regarding the clinical merits of universal screening [7–13]. Those who support universal screening in health care settings argue that effective screening tools are available [9], screening increases IPV detection rates [14, 15], and the majority of women view screening as acceptable provided that it is conducted in private, sensitive, and non-judgmental manner [16, 17]. Those who do not support universal screening argue that there is insufficient evidence to support implementation [7, 11] and that potential adverse outcomes from such programs are unknown [8]. Existing guidelines vary in their recommendations regarding IPV screening and identification practices (e.g. [18–22]). This lack of consensus has resulted in additional research and systematic reviews attempting to provide clear recommendations, however the merits of universal screening and the optimal approach to IPV identification remain contentious. Given the controversies faced and the vast number of studies on the topic, we conducted a scoping review to broadly identify and synthesize the available literature evaluating IPV identification/screening programs within health care settings in order to identify areas for potential evidence-based recommendations and to focus research priorities in the field. Given the breadth and diversity of the existing IPV identification program literature available, a scoping review is the most appropriate methodology to address our research objectives.

## Materials and Methods

Following the scoping review framework proposed by Arksey and O’Malley [23], we used an integrated research process to obtain knowledge user input throughout all six stages of the review’s methodology. Knowledge users are defined as those who are “likely to be able to use research results to make informed decisions about health policies, programs and/or practices” [24]. A collaboration of physicians, HCPs, researchers, IPV advocates, and IPV victim representatives (see Acknowledgments) made up the knowledge users for our scoping review and directed our research goals and methodology.

### Literature Search Strategy

We consulted with a biomedical librarian to develop a sensitive search strategy to identify all types of publications involving IPV identification, assistance, and educational programs in health care settings within the published literature. Several search strategies and sources were used to identify relevant studies. We used a combination of keywords and medical subject heading (MeSH) terms related to IPV, to search the following electronic databases: MEDLINE, Embase, Cumulative Index of Nursing and Allied Health Literature (CINAHL), Cochrane Database of Systematic Reviews (CDSR), Cochrane Central Register of Controlled Trials (CENTRAL), and psycINFO. All searches were performed in July 2015 and the search was limited to articles published from 2000 and onwards. No language restrictions were employed. Additionally, we conducted a hand search of systematic reviews, meta-analyses, and recently published included studies. A sample of the electronic search strategy is outlined in **S1 Table**.

### Eligibility Criteria

We included studies in this scoping review if they met the following broad eligibility criteria: (1) published in English; (2) published in full-text format; (3) focused on IPV; (4) evaluated an IPV identification program or IPV screening program for women in a health care setting; (5) level I to IV evidence or used qualitative research methodology; and (6) population comprised of adults. Systematic reviews and meta-analyses were included if they otherwise met our eligibility criteria. We excluded studies that described an IPV screening program but did not evaluate it as well as studies in which an identification program was not the primary intervention (e.g. studies in which IPV assistance or educational programs were the primary intervention evaluated). We also excluded narrative reviews and studies that were published as dissertation abstracts or conference proceedings.

### Article Selection

We reviewed titles of all references identified in the literature search independently and in duplicate (S.B., A.H., K.T., and T.S.). We also reviewed abstracts of all references identified as potentially eligible during title screening independently and in duplicate (E.A., S.B., A.H., M.M., A.S., H.S., S.S., and T.S.). During title screening and abstract screening, reviewers erred on the side of inclusion and included any title that may have potentially met the eligibility criteria. Any conflicts between reviewers about whether or not a title was potentially eligible resulted in inclusion at this stage of the selection process. Two reviewers (S.S. and T.S.) independently and in duplicate reviewed the full-text articles of all references included at the abstract screening level. Any conflicts between the two reviewers were discussed until consensus was reached. All article screening was completed using the web-based program DistillerSR.

### Data Extraction

We completed data extraction for all included studies. Briefly, we extracted data related to study characteristics (e.g. location of research, year of publication, type of journal, etc.), study design characteristics (e.g. study design, number of participants, etc.), screening program characteristics (e.g. form of screening, HCP conducting screening, number of times screening conducted, etc.), methodological characteristics (e.g. use of control group, follow-up, drop-out rate, etc.), and its evaluation (i.e. outcome measure themes). Additionally, studies were classified based on program evaluation results as determined by author conclusions (i.e. positive vs. not positive). Two reviewers independently completed the data extraction. Any disagreements in data extraction were resolved by a third reviewer. We completed data extraction in Distiller SR using pre-designed data extraction forms which were piloted to ensure all key information was captured. We provided an instruction manual to each reviewer detailing instructions for data extraction to ensure consistency and accuracy of the extracted data.

### Data Analysis

We used descriptive statistics to summarize all data. For continuous data, we reported the mean and standard deviation or median and interquartile range (IQR) based on the distribution of the data. We used counts and proportions to describe all other data. No inferential statistical testing was performed.

## Results

### Article Identification and Selection

**Fig 1:**
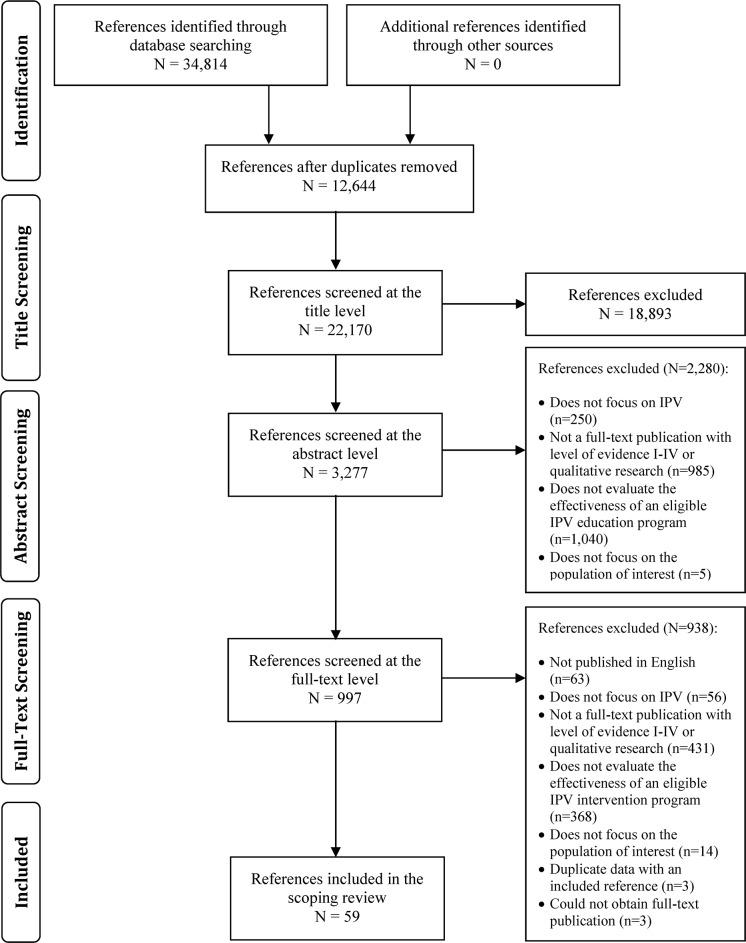
Literature Search Results and Study Selection.

Our search strategy identified a total of 34,814 articles (Fig 1). Of these, 12,644 were duplicates references and were removed prior to our title screening. Therefore, we reviewed 22,170 titles for eligibility and found that 3,277 articles were potentially eligible. Our abstract screening identified 997 potentially eligible abstracts for which we reviewed the full-text. Following the full-text review, we identified 59 articles that met the eligibility criteria and were included in this scoping review [8–10, 12–15, 17, 25–75]. Our hand search of systematic reviews and recently published included studies did not identify any additional eligible studies.

### Study Characteristics

The majority of research was conducted in North America (56%, n = 33), Australia/Oceania (25%, n = 15) and the United Kingdom (12%, n = 7) (Table 1). Only a small proportion of the studies were conducted in Asia (5%, n = 3), Continental Europe (5%, n = 3), and Africa (2%, n = 1) and no research was conducted in South America. Nearly half (42%, n = 25) of all studies were published in medical journals with 19 percent (n = 11) published in nursing journals and 15 percent (n = 9) in women’s health or IPV journals. Less than 25 percent of studies were published in other types of journals. Three-quarters of studies were funded (75%, n = 44). Of these 44 studies, government funding was the most frequent (66%, n = 29) followed by foundation, association, or other non-profit funding (23%, n = 10). Twenty percent (n = 12) of studies did not specify if funding was received and five percent (n = 3) were unfunded.

**Table 1:** Study Characteristics.

Characteristic	Frequency
	N(%) N = 59
Location of Research*	
North America	33 (55.9)
Australia/Oceania	15 (25.4)
United Kingdom	7 (11.9)
Asia	3 (5.1)
Continental Europe	3 (5.1)
Africa	1 (1.7)
**Year of Publication**	
2000–2004	13 (22.0)
2005–2009	25 (42.4)
2010–2015	21 (35.6)
**Type of Journal**	
Medical Journal	25 (42.4)
Nursing Journal	11 (18.6)
Women’s Health or IPV Journal	9 (15.3)
Midwifery Journal	7 (11.9)
Social Science Journal	5 (8.5)
Health Services/Health Policy Journal	1 (1.7)
Sexual Health Journal	1 (1.7)
**Study Funding**	
**Funded (n = 44)**	
Government	29 (65.9)
Foundation/Association/Non-Profit	10 (22.7)
Government and Foundation/Association/Non-Profit	4 (9.1)
Government + Foundation/Association/Non-Profit + Industry/Corporate/Profit	1 (2.3)
Not Specified	12 (20.3)
Unfunded	3 (5.1)

*Numbers do not sum to 59 and percentages do not sum to 100 as some studies were conducted in multiple locations.

### Study Design and Methodological Characteristics

The included studies used a variety of study designs including randomized controlled trials (19%, n = 11), qualitative (n = 8), cross-sectional (n = 8), and mixed-method (n = 8) study designs (Table 2). Three-quarters (n = 44) of studies reported the number of centres participating in the studies. Of these 44 studies, the majority (59%, n = 26) were single centre initiatives, 23 percent (n = 10) included two to four centres, nine percent (n = 4) included five to nine centres, and an additional nine percent (n = 4) included ten or more centres. The median number of participants in the included studies was 302 (1^st^ quartile: 126; 3^rd^ quartile 1,281). The number of participants ranged from 5 [71] to 35,613 [8]. Four studies included more than 10,000 participants and were identified as outliers [8, 10, 47, 57]. Three of these studies were systematic reviews [8, 10, 47] and one was a case series [57]. When these outliers are removed, the mean number of participants across all included studies was 852.4 (SD = 1,451.7).

**Table 2:** Study Design and Methodological Characteristics.

Characteristic	Total	Reference
	N(%) N = 59	
**Study Design**		
Randomized Controlled Trial	11 (18.6)	12,13,26,28,30,37,38,39,40,43,52
Qualitative Study	8 (13.6)	17,35,41,56,59,64,66,73
Cross-Sectional Study	8 (13.6)	36,54,58,60,61,67,69,75
Mixed-Methods Study	8 (13.6)	25,27,42,49,50,71,72,74
Systematic Review	7 (11.9)	8,9,10,14,15,46,51
Case Series	6 (10.2)	33,34,44,48,53,57
Pre-Test/Post-Test	5 (8.5)	29,31,63,68,70
Prospective Comparative Study	4 (6.8)	32,45,55,62
Meta-Analysis	1 (1.7)	47
Retrospective Comparative Study	1 (1.7)	65
Number of Centres (n = 44)*		
1	26 (59.1)	13,26,27,29,31,32,34,36,37,39,40,42,44,45,48,49,50,53,55,62,68–72,75
2–4	10 (22.7)	17,28,30,41,52,54,57,60,61,63
5–9	4 (9.1)	43,59,64,67
≥10	4 (9.1)	12,38,56,58
**Study Setting‡**		
Obstetrics/Gynecology	26 (44.1)	8–10,12,14,15,17,27,29,36–39,46–49,51,53,56–58,60,61,66,67
Emergency Department	21 (35.6)	8–10,12,14,32,34,40,41,43,45,46,47,50,51,52,60–62,72,75
Family Medicine	15 (25.4)	8–10,12–14,17,28,43,46,47,51,64,68,70
Community Health Centre	8 (13.6)	8,9,12,38,41,46,51,71
Family Planning Clinic	5 (8.5)	9,38,39,54,63
Home Visiting Program	5 (8.5)	17,31,33,35,65
Midwifery	5 (8.5)	25,36,59,73,74
Pediatrics	5 (8.5)	10,14,26,46,57
Mental Health	4 (6.8)	46,56–58
Internal Medicine	2 (3.4)	14,55
Sexual Health Clinic	2 (3.4)	44,46
Women’s Health Clinic	2 (3.4)	30,43
Abortion Clinic	1 (1.7)	69
Outpatient Hospital Department	1 (1.7)	42
Number of Participants (median (1^st^ Q, 3^rd^ Q))		302 (126, 1281)
**Use of Control/Comparative Group(n = 51)†**		
Yes	26 (51.0)	12,13,25–32,37–40,43,45,52,55,60,62,63,65,68,70,72,74
**Inclusion of a Follow-Up Period (n = 51)†**		
Yes	13 (25.5)	12,17,27,29,31,33,37–40,63,68,74
**Length of Follow-up (months) (n = 13)**		
<0 to ≥3	3 (23.1)	27,39,40
<3 to ≥6	4 (30.8)	33,37,63,74
<6	3 (23.1)	12,38,68
Not Reported	3 (23.1)	17,29,31
**Dropout Rate (n = 13)**		
0	2 (15.4)	29,68
1 to 30	6 (46.2)	37–40,63,74
>30	2 (15.4)	12,27
Not Reported	3 (23.1)	17,31,33
**Outcome Themes (n = 51)**		
IPV Disclosure	35 (68.6)	12,13,26–28,30–34,36,37,39,41–45,48–50,52–54,57,58,60–62,65,68,69,71,74,75
Screening	20 (39.2)	25,29,36,44,48–50,53–55,57,59,62,63,65,69–71,74,75
HCP Opinions Towards Screening	19 (37.3)	13,17,28,35,36,42,44,49,50,54,56,59,61,63,66,71–74
Patient Opinions Towards Screening	15 (29.4)	13,17,26–28,36,37,39,41,43,50,58,64,67,71
Barriers to Screening/Disclosure	12 (23.5)	25,35,49,50,56,59,64,69,71,73–75
Use of IPV Resources	10 (19.6)	12,17,36,38,39–41,62,63,75
Referral to IPV Services	8 (15.7)	13,33,50,52,62,63,72,75
IPV Discussions with HCP	5 (9.8)	13,17,50,52,63
Chart Documentation of IPV	4 (7.8)	13,49,63,65
IPV Severity/Recurrence	3 (5.9)	12,38,40
Screening Form Completion	3 (5.9)	43,54,60
Screening Harm Measures	3 (5.9)	12,41,58
Substance Use/Abuse	3 (5.9)	12,13,37
Health/Wellbeing	2 (3.4)	12,38
Economic/Efficiency	2 (3.4)	17,28
Safety	2 (3.4)	13,40
Other	5 (9.8)	25,38,42,58,75

*Number of centres was either not applicable or not reported for 15 studies.

‡Numbers do not sum to 59 and percentages do not sum to 100 as some studies reported multiple characteristics.

†Data not abstracted for 7 systematic review and 1 meta-analysis studies.

Half of all studies (n = 26) included a control or comparative group and one quarter (n = 13) included a follow-up period. Of the 13 studies that included a follow-up period, 23 percent (n = 3) followed participants for up to three months, 31 percent (n = 4) followed participants for three to six months, and 23 percent followed participants for more than 6 months. Length of follow-up was not clearly reported for an additional 23 percent of studies (n = 3). Additionally, of the 13 studies that reported a follow-up period, 15 percent (n = 2) of studies reported a zero percent dropout rate, 46 percent (n = 6) reported a dropout rate of less than 30 percent, and 15 percent (n = 2) reported a dropout rate greater than 30 percent. The dropout rate for 23 percent (n = 3) of studies was not clearly reported.

Studies reported 16 different outcome themes (Table 2). The most commonly reported outcome theme was IPV disclosure which was reported in 69 percent (n = 35) of studies. This was followed by number of patients screened (39%, n = 20), HCP opinions towards screening (37%, n = 19), patient opinions towards screening (29%, n = 15), barriers to screening or disclosure (24%, n = 12), use of IPV resources (19.6%, n = 10), and referral to IPV services (16%, n = 8). The additional nine outcomes themes were reported in less than ten percent of studies. Three different studies [12, 38, 40] reported outcomes pertaining to improvements in women’s lives (i.e. IPV severity/recurrence (n = 3, 5.9%) and health/wellbeing (n = 2, 3.4%)).

### Intervention Characteristics

Identification interventions took place in 14 different health care settings. The most frequent setting was obstetrics and gynecology (44%, n = 26), followed by emergency medicine (36%, n = 21), family medicine (25%, n = 15), and community health centres (14%, n = 8). Three quarters (n = 38) of studies evaluated IPV identification programs that involved in-person screening (Table 3). Fourteen percent (n = 7) examined computer-based screening, ten percent (n = 5) paper-based, and two percent (n = 1) audio/visual based. In-person identification interventions were performed by a variety of different HCPs and study personnel. Nurses were the most common group (45%, n = 17) followed by midwives (24%, n = 9), social workers, counsellors, or IPV coordinators (13%, n = 5), and physicians or surgeons (11%, n = 4). Other HCPs and study personnel conducted screening in less than six percent of studies. Eighty percent (n = 41) of studies specified that HCPs were provided with some sort of IPV identification training. Approximately three quarters of the studies specified the number of times women were screened with the majority (63%, n = 32) being screened once.

**Table 3:** Intervention Characteristics.

Characteristic	Total	Reference
	N(%) N = 51*	
**IPV Identification Intervention**		
In-person	38 (74.5)	17,25,29–36,40–42,44,45,48–50,53–59,61,63–67,69–75
Computer	7 (13.7)	13,27,38,39,43,52,62
Paper	5 (9.8)	12,28,37,60,68
Audio/video	1 (2.0)	26
**HCP who Performed Screening (n = 38)**		
Nurse	17 (44.7)	31,33,35,40–42,49,50,53–55,61,63,65,66,72,75
Midwife	9 (23.7)	17,25,36,40,59,61,67,73,74
Social Worker/Counsellor/IPV coordinator	5 (13.2)	33,34,40,53,69
Physician/Surgeon	4 (10.5)	42,50,53,70
Study Personnel	3 (7.9)	17,30,45
Other Allied HCPs	3 (7.9)	33,42,71
Resident	1 (2.6)	29
Not Specified	7 (18.4)	32,44,48,56–58,64
**Identification Training Provided to HCPs**		
Yes	41 (80.4)	12,13,17,25,26,28–31,33,35–38,40–44,48–50,53,54,56–63,65,67–72,74,75
No	3 (5.9)	27,52,73
Not Specified	7 (13.7)	32,34,39,45,55,64,66
**Number of Times Women Were Screened**		
Once	32 (62.7)	12,13,26–28,30–32,34–36,38,45,50,52,53,56,58,60–62,67–70,72
Three times	4 (7.8)	17,29,37,59
Every three months	1 (2.0)	49
Not specified	14 (27.5)	25,33,48,54,55,57,63–66,71,73–75
**Questionnaire Used for Identification†**		
Yes	37 (72.5)	12,13,17,26–28,30,32,33,36–45, 49,50,52,54,56–62,64,65,67–69,71,72
No	14 (27.5)	25,29,31,34,35,48,53,55,63,66,70,73–75
**Questionnaire Used (n = 10)†**		
Partner Violence Screen	4 (40.0)	32,38,39,43
Woman Abuse Screening Tool	4 (40.0)	12,28,43,60
Hurt, Insult, Threaten, And Scream	2 (20.0)	28,64
Composite Abuse Scale	1 (10.0)	43
Conflict Tactics Scale	1 (10.0)	30
Violence Against Women Screen	1 (10.0)	37
**Assistance Provided to Women Experiencing IPV**		
Yes	33 (64.7)	12,13,17,26,28,30,33,34,36–45,48–50,54–59,62–64,71,72,75
Not Specified	18 (35.3)	25,27,29,31,32,35,52,53,60,61,65–70,73,74
**Type of Assistance (n = 33)†**		
IPV Resources	28 (84.8)	12,13,17,26,28,30,33,34,36–39,41,43–45,50,54–59,62,64,71,72,75
Referral	18 (54.5)	13,33,34,36,37,40–42,45,49,50,54,56,58,62,63,71,72
Counselling/Advocacy	14 (42.4)	13,17,33,34,37–39,42,45,48,50,55,63,72
Safety Planning	8 (24.2)	13,33,34,37,40,41,62,63
Home Visitation	2 (6.1)	17,33

*Data not abstracted for 7 systematic review and 1 meta-analysis studies.

†Percentages do not sum to 100 as some studies reported multiple intervention characteristics.

Three-quarters of studies used a questionnaire for IPV identification. Thirty percent (n = 15) of studies developed their own questionnaire for the study while 26 percent (n = 13) adapted an existing questionnaire and 20 percent (n = 10) used an existing questionnaire. Of the ten studies that used an existing questionnaire, the most commonly used questionnaires were the Partner Violence Screen (PVS) (40%, n = 4) and the Women Abuse Screening Tool (WAST) (40%, n = 4). Two-thirds of studies (n = 33) specified that some sort of assistance was provided to women who screened positive for IPV. The types of assistance provided included provision of IPV resources (85%, n = 28), referral (55%, n = 18), counselling/advocacy (42%, n = 14), safety planning (24%, n = 8), and home visitations (6%, n = 2).

### Studies Reporting Positive Program Evaluation Results

Our scoping review identified 36 studies (70.6%) that reported positive program evaluation results, 10 studies (19.6%) that reported neutral or mixed results, 4 studies (7.8%) where the results were not concluded, and 1 study (2.0%) where the results were negative. We looked at outcome themes by stratifying all studies by program evaluation (i.e. positive versus not positive). Outcome themes amongst studies frequently reporting positive results (i.e. positive results reported for ≥75% of studies in which at least 5 studies include the specified program characteristic) included those that looked at outcomes pertaining to screening (80.0% of studies reported a positive program evaluation) and IPV disclosure (77.1% of studies reported a positive program evaluation) (Table 4). Of the three studies that reported outcomes assessing improvements in women’s lives (i.e. IPV severity/recurrence and health/wellbeing), all three reported neutral program evaluation results. Of the 48 studies that did not report outcomes assessing improvements in women’s lives, 36 (75.0%) reported positive program evaluation results, 7 (14.6%) reported neutral or mixed results, 4 (8.3%) did not specify program evaluation conclusions, and 1 (2.1%) reported negative results.

**Table 4:** Outcome Theme by Program Evaluation Results.

Outcome Theme	Total	Frequency	
		Total%	
	N(%) N = 51*	Positive N(%)	Not Positive N(%)
		N = 36 (70.6%)	N = 15 (29.4%)
IPV Disclosure	35 (68.6)	27 (77.1)	8 (22.9)
Screening	20 (39.2)	16 (80.0)	4 (20.0)
HCP Opinions Towards Screening	19 (37.3)	11 (57.9)	8 (42.1)
Patient Opinions Towards Screening	15 (29.4)	11 (73.3)	4 (26.7)
Barriers to Screening/Disclosure	12 (23.5)	7 (58.3)	5 (41.7)
Use of IPV Resources	10 (19.6)	6 (60.0)	4 (40.0)
Referral to IPV Services	8 (15.7)	6 (75.0)	2 (25.0)
IPV Discussions with HCP	5 (9.8)	2 (40.0)	3 (60.0)
Chart Documentation of IPV	4 (7.8)	3 (75.0)	1 (25.0)
IPV Severity/Recurrence	3 (5.9)	0 (0.0)	3 (100.0)
Screening Form Completion	3 (5.9)	2 (66.7)	1 (33.3)
Screening Harm Measures	3 (5.9)	2 (66.7)	1 (33.3)
Substance Use/Abuse	3 (5.9)	2 (66.7)	1 (33.3)
Health/Wellbeing	2 (3.4)	0 (0.00)	2 (100.0)
Economic/Efficiency	2 (3.4)	0 (0.0)	2 (100.0)
Safety	2 (3.4)	1 (50.0)	1 (50.0)
Other	5 (9.8)	4 (80.0)	1 (20.0)

*Data not abstracted for 7 systematic review and 1 meta-analysis studies.

## Discussion

This scoping review represents a comprehensive overview of the published literature on IPV identification programs and IPV screening programs within health care settings. Our review included 59 studies evaluating different IPV identification programs within health care settings. The majority of this research was conducted in North America (56%) and Australia/Oceania (25%). Only a small proportion of the studies were conducted in Asia, Continental Europe, and Africa and no research was conducted in South America. This may represent differing attitudes towards IPV and IPV identification within health care settings internationally. IPV identification studies were published in a variety of different journals indicating an interest in IPV identification research across multiple health care disciplines.

A wide variety of study designs were used within the IPV identification literature with randomized controlled trials being the most common design (19%) followed by qualitative, cross-sectional, and mixed methods studies (14% each). The frequent use of qualitative and mixed methods study designs likely reflects the importance of rich data describing patient and HCP insight into IPV identification. For example, qualitative and mixed methods studies have focused on understanding women’s perceptions and experiences with undergoing IPV screening [17, 27, 41] as well as HCPs’ experiences, perceptions, and comfort with conducting screening [49, 56, 59, 66].

Only one-quarter of all studies, and 45% of all RCTs, included a follow-up period. The limited number of studies that chose to include a follow-up period is likely explained by the type of outcomes measured. For example, outcomes such as screening rate, IPV disclosure rate, patient opinions towards screening (which were three of the four most commonly reported outcomes) do not require follow-up periods. Conversely, other outcomes such as use of IPV resources, health and wellbeing, or IPV severity or recurrence require follow-up periods to assess changes in outcomes, however these outcomes were assessed less frequently. Coster [76] emphasizes the importance of ensuring that study follow-up times are selected so that they are long enough to allow for changes to be seen in the construct being measured, and for measurement instruments to detect the changes. Therefore, the heterogeneity in outcome measures within the included studies may partially explain the variation in the inclusion of a follow-up period and follow-up period duration.

Interventions took place in a number of different health care settings indicating an interest and need for IPV identification programs across multiple settings. While programs included substantial heterogeneity, the majority of studies reported positive results for IPV screening programs (71% of studies). This suggests that the most important factor may be that women are being asked about IPV in some manner, and the details about how they are asked are less important.

Over 16 different outcome themes were identified across the included studies. The most common outcome themes focused on determining whether screening took place (e.g. screening rate), whether women disclosed the occurrence of IPV (e.g. disclosure rate), and patients’ and HCPs’ feelings towards the identification program. Studies that included screening and IPV disclosure outcomes themes frequently reported positive program evaluation results (80.0% and 77.1% respectively). While these outcomes are important in order to assess if the identification program is feasible (i.e. ability to implement the program and have it accepted by women and HCPs) and accomplishing the primary goal of identifying women who are experiencing IPV, they are not able to assess whether or not women were actually helped. Three different RCTs included in our scoping review did assess these types of outcomes which included: IPV severity or recurrence (3 studies) and health and wellbeing (2 studies). The programs being evaluated in these studies included varying forms of assistance for women including IPV resource lists, statements about the unacceptability of violence, risk assessment, and referral. Of the three studies that assessed IPV severity and recurrence, two [38, 40] reported no significant differences between participants in the IPV identification group and the control group and one reported small differences in favour of the IPV identification group [12]. Neither of the two studies that assessed health and wellbeing outcomes reported differences between the IPV identification group and control group [12, 38].

These same three studies [12, 38, 40] all three reported neutral program evaluation results. This is in contrast to the 48 studies that did not include outcomes assessing improvements in women’s lives, which reported positive program evaluation results 75 percent of the time (n = 36). Although the sample size is small, these findings suggest that conclusions regarding program evaluation may be influenced by the outcomes selected. For example, given the complex and multifaceted nature of IPV, it is unlikely that identification programs alone (i.e. those that are not also coupled with assistance and educational programs) will result in reductions in the severity/recurrence of IPV or improvements in health/wellbeing outcomes. This is because the purpose of identification program is simply to determine which women are experiencing IPV and consequently, their ability to lead to improvement women’s circumstances is dependent on the provision of effective assistance interventions by qualified HCPs. Future research should focus on assessing patient important outcomes in IPV programs that include all screening, assistance, and educational components with particular attention paid to the impact of outcome selection on result interpretation.

This scoping review has several strengths that contribute to its quality. A research librarian with expertise in the area designed and conducted the search strategy to ensure all published literature was captured. Additionally, all article screening was completed in duplicate by reviewers with both content and methodological expertise. Finally, to the best of our knowledge this scoping review is the first to assess IPV identification programs.

Despite these strengths, our scoping review is limited by the restriction of our search to published literature. This may introduce publication bias into our results as it is possible that there are a higher proportion of studies with neutral or negative results that are unpublished compared to positive ones. Our scoping review was also limited to studies published in English which may partially explain the limited number of studies found that were conducted in Asia, Continental Europe, South America, and Africa. Additionally, the heterogeneity of study design and interventions introduced challenges of capturing all of the details of each included study. However, by focusing our scoping review on the commonalities between studies we were able to produce a comprehensive summary of the existing literature.

## Conclusions

Overall, the results of the current scoping review provide a comprehensive summary of the existing literature in the field of IPV identification programs within health care settings. The majority of studies included in this scoping review reported positive program evaluation results. This may suggest that many different IPV identification programs are beneficial, particularly in regards to identifying victims of abuse; however, it remains unknown as to whether identification programs prevent future episodes of abuse. Additionally, the substantial heterogeneity of the intervention characteristics, study methodology, and outcome measures assessed limits the ability to make clear recommendations as to the optimal method(s) of screening.

## Supporting Information

S1 Database Minimal Data Set. (XLSX)

S1 Table Search Strategy (e.g. MEDLINE). (DOCX)

S2 Table PRISMA Checklist. (DOCX)
