# Supplementary material for: A Scoping Review of Intimate Partner Violence Screening Programs for Health Care Professionals
Source: PLoS One. 2016 Dec 15;11(12):e0168502. doi: 10.1371/journal.pone.0168502 (PMC5158065; doi:10.1371/journal.pone.0168502)
Supplement: S1 Table — (DOCX) [file pone.0168502.s002.docx]

**S1 Table:** Search Strategy (e.g. MEDLINE)

Database: Ovid MEDLINE(R) In-Process & Other Non-Indexed Citations, Ovid MEDLINE(R) Daily and Ovid MEDLINE(R) <1946 to Present>

| 1 | spouse abuse.mp. or exp Spouse Abuse/ |
| --- | --- |
| 2 | (intimate partner adj4 (abus* or aggression or sexual or violence or victimization or victim*)).mp. |
| 3 | (partner adj4 (abus* or aggression or violence or victimization or victim*)).mp. (5153) |
| 4 | (domestic partner adj4 (abus* or aggression or sexual or violence or victimization or victim*)).mp. |
| 5 | (intimate adj2 (violence or victim:)).mp. |
| 6 | (woman abuse or battered spouse).mp. |
| 7 | (spousal adj (abuse or violence)).mp. |
| 8 | (woman abuse or battered spouse or battered woman).mp. |
| 9 | ((wife or spouse or spousal or partner) adj battering).mp. |
| 10 | wife rape.mp. |
| 11 | ((spousal or spouse) adj rape).mp. |
| 12 | batterer.mp. |
| 13 | ((spouse or domestic or wife or spousal or woman) adj (abuse or abuser)).mp. |
| 14 | couple violence.mp. |
| 15 | or/1-14 |
| 16 | battered women.mp. or Battered Women/ |
| 17 | battering.mp. |
| 18 | domestic violence.mp. or Domestic Violence/ |
| 19 | family violence.tw. |
| 20 | violence against women.mp. |
| 21 | or/16-20 |
| 22 | ((risk or experienc:) adj3 (abuse or violence: or abusive)).mp. |
| 23 | Mass Screening/ |
| 24 | Risk Assessment/ |
| 25 | health facilities/ or exp academic medical centers/ or ambulatory care facilities/ or exp outpatient clinics, hospital/ or pain clinics/ or surgicenters/ |
| 26 | Emergency Service, Hospital/ or exp hospitals/ |
| 27 | education.ti,kw. |
| 28 | (perpetrator: or abuser:).mp. |
| 29 | "interprofessional education".kw. |
| 30 | (assessment or screening).ti,kw. |
| 31 | training.mp. |
| 32 | hospital*.ti. |
| 33 | or/22-32 |
| 34 | 21 and 33 |
| 35 | 15 or 34 |
| 36 | limit 35 to yr="2000 -Current" |
